# Self-righting potential and the evolution of shell shape in Galápagos tortoises

**DOI:** 10.1038/s41598-017-15787-7

**Published:** 2017-11-30

**Authors:** Ylenia Chiari, Arie van der Meijden, Adalgisa Caccone, Julien Claude, Benjamin Gilles

**Affiliations:** 1 University of South Alabama, Department of Biology, Mobile, AL 36688 USA; 2 CIBIO/InBIO, Centro de Investigação em Biodiversidade e Recursos Genéticos da Universidade do Porto, Campus Agrário de Vairão, 4485-661 Vairão, Portugal; 3 Department of Ecology and Evolutionary Biology, Yale University, New Haven, CT 06520 USA; 4 Institut des Sciences de l’Evolution, CNRS-UMR n° 5554, CC 064, Université de Montpellier, 2, Place Eugène Bataillon, 34095 Montpellier, cedex 5 France; 5 Laboratoire d’Informatique, de Robotique et de Microélectronique de Montpellier, CNRS-UMR n° 5506, CC477, Université de Montpellier, 161 rue Ada, 34095 Montpellier, Cedex 5 France

## Abstract

Self-righting, the capacity of an animal to self-turn after falling on its back, is a fitness-related trait. Delayed self-righting can result in loss of mating opportunities or death. Traits involved in self-righting may therefore be under selection. Galápagos giant tortoises have two main shell morphologies - saddleback and domed – that have been proposed to be adaptive. The more sloped shape on the sides of the shell and the longer extension of neck and legs of the saddlebacks could have evolved to optimize self-righting. The drier environments with more uneven surfaces where the saddleback tortoises occur increases their risk to fall on their back while walking. The ability to fast overturn could reduce the danger of dying. To test this hypothesis, we used 3D shell reconstructions of 89 Galápagos giant tortoises from three domed and two saddleback species to compare self-righting potential of the two shell morphotypes. Our results indicate that saddleback shells require higher energy input to self-right than domed ones. This suggests that several traits associated with the saddleback shell morphology could have evolved to facilitate self-righting. Studying the functional performances of fitness-related traits, as in this work, could provide important insight into the adaptive value of traits.

## Introduction

Self-righting, the capacity of an animal to self-turn after falling on its back, is a fitness-related trait for terrestrial animals. Animals can fall on their backs due to locomotion on uneven surfaces, conspecific interactions such as fighting, predator encounters, or from falling through the air in the case of flying insects^1–6^. Delayed self-righting can result in loss of mating opportunities or death due to desiccation, predation, starvation, or hampered breathing^7–10^. Variation in self-righting strategy and performance – how quickly an animal turns itself over – depends on the flexibility of the body and body shape (reviewed in^3^). Particularly, in animals with rigid and armored bodies such as crustaceans, some insects, and turtles, the feet generally cannot touch the ground when they are on their backs and self-righting is determined by body shape, body size, and extension or length of movable body parts (*e*.*g*., neck and legs) that help create momentum for the animal to roll over^3,10–12^.

**Figure 1:**
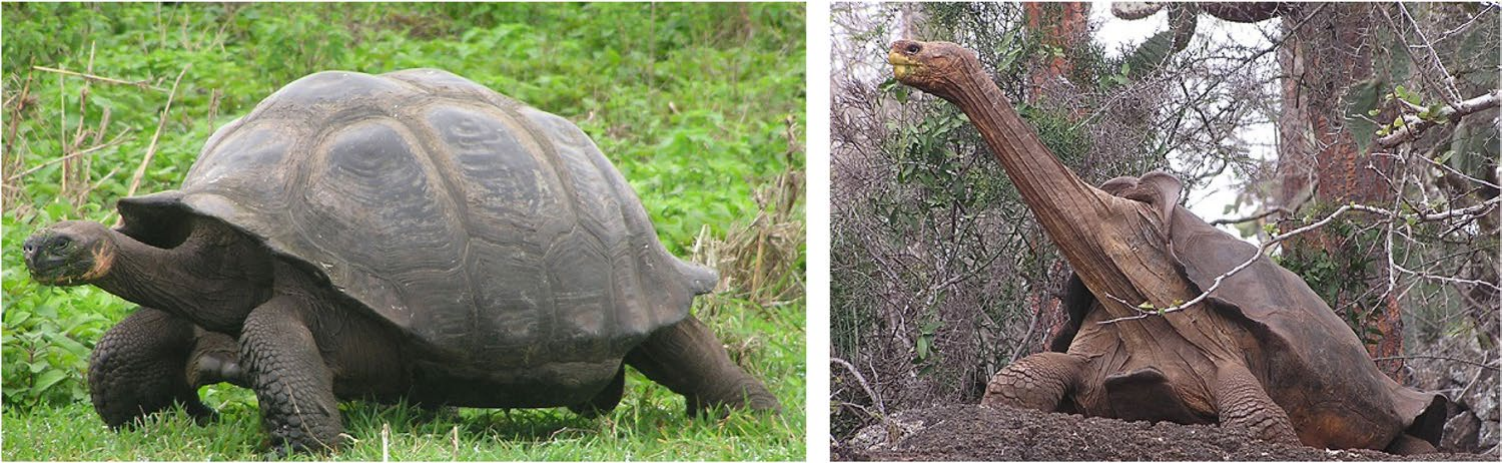
Saddleback (right) and domed (left) shell morphotypes in Galápagos giant tortoises. Photos by Y. Chiari.

In turtles, shell morphology determines the self-righting strategy used and its performance: turtles with flatter shells use a combination of vertical push given by the neck and waving of the legs to gain momentum to turn, while turtles with more domed shells mostly rely on waving their legs^11^. Among turtles, Galápagos giant tortoises offer an ideal system for examining how differences in the rigid body shape (shell morphotypes) may influence self-righting performance, and thus survival and fitness. In this species group, shell morphology varies extensively both within and among species and islands. Galápagos giant tortoises comprise multiple species with two main distinct shell morphologies, saddleback and domed that have evolved multiple times in the archipelago, with some species being either clearly domed or saddleback, while others have intermediate shell shapes^13,14^. Saddleback shells have a higher anterior opening, which allows for higher extension of the neck, and a more compressed carapace on the sides, while domed tortoises have a cupula-like carapace^15,16^ (Fig. 1).

The different Galápagos giant tortoise shell morphotypes generally occur in different habitats, with saddleback tortoises occupying drier and lower elevation environments, while domed tortoises are found in more humid, colder and higher elevation habitats^17–21^. Although a correlation between shell morphotype and habitat type has been reported in Galápagos giant tortoises^19–21^, its existence does not necessarily imply evolutionary causation, as habitat selection may be a consequence rather than a cause of shell shape evolution.

The two shell morphotypes have been proposed to be adaptive for distinct feeding niches (*e*.*g*.,^13,17–23^). However, there are currently no data supporting this hypothesis. Vegetation is more luxuriant where domed tortoises occur, while it is drier and with more cacti where saddleback tortoises live^17–19^. Other selective forces, such as different self-righting performance between saddleback and domed tortoises may also be considered as a driver of shell shape variation in these animals. Galápagos giant tortoises commonly walk on irregular surfaces and often fall on their back or in crevices between lava rocks; delayed self-righting may therefore increase their chance of mortality (E. Garcia, pers. comm.). In drier lower elevation environments, where saddleback tortoises occur, walkable surfaces are mostly uneven, consisting largely of jagged lava rocks (*e*.*g*., Espanola and Pinzon Islands^24^) and temperatures generally higher. Saddleback tortoises therefore have a higher risk of falling on their backs (E. Garcia, pers. comm.), thus, quick self-righting capacity would prevent the animals from dying due to desiccation or starvation. The larger neck extension capacity in saddlebacks as compared to domed tortoises^25^, together with the more compressed sides of the shell of the saddlebacks, could facilitate successful turning.

Although the adaptive role of different shell morphotypes in Galápagos giant tortoises in the use of feeding resources, self-righting, or other functions (*e*.*g*., thermoregulation) is compelling, it has not been yet formally tested. In this work, we test the differential energy requirement to potentially successfully self-right for tortoises with saddleback or domed shell morphotypes. In this work, we focus on understanding the influence of the different shell morphologies on self-righting – *i*.*e*., excluding the contribution of the neck or limb movement to it. Therefore, we reconstructed in 3D the shell of 89 domed and saddleback Galápagos giant tortoises and inferred which shell morphotype would require a higher energy input from the animal to successfully get back on its feet after falling on its back. Because of the higher risk of falling on their back and its implication for fitness, we expected saddleback tortoises to self-right more easily than domed ones.

## Materials and Methods

### Ethics statement

Experiments carried out on the two live animals took place at the Rotterdam Zoo. Experiments were carried out following directions of the zoo staff and according to guidelines and regulation of the EAZA (European Association for Zoos and Aquaria). No additional permits were required. All experiments were performed in accordance with relevant guidelines and regulations.

### Data collection

To obtain shell shape data for saddleback and domed Galápagos giant tortoises, 89 sexually mature individuals of both sexes (57 domed and 32 saddleback tortoises) belonging to five different species were sampled in the field (*Chelonoidis porteri* and *C*. *donfaustoi*, both domed, and *C*. *hoodensis*, saddleback) and at the California Academy of Sciences (*C*. *hoodensis* and *C*. *ephippium*, saddleback and *C*. *vicina*, domed; Supplementary Table S1) at different times. Datasets for *C*. *porteri* and *C*. *donfaustoi* are subsets of the data used in^16,26^, without including the juveniles and the individuals of uncertain species assignment (see^26^). Sampling for 3D carapace reconstructions followed^16,26,27^. Briefly, digital images (10–15 per individual) of the carapace were obtained with a camera (see^16,26,27^ for camera models and resolution, and accuracy of the reconstructions). 3D reconstructions were carried out with PhotoModeler Pro 5.2.3 (Eos Systems Inc.) and reconstructed carapaces were scaled to the actual animal size following^16,26^. Coordinates of the 25 landmarks used for the carapace 3D reconstructions of each individual are provided in Supplementary Table S1.

Depending on their different shell morphotypes, turtles use different strategies to self-right with more or less involvement of the neck, head and legs^11^. To evaluate if saddleback tortoises have effectively longer necks than domed individuals or if the neck can only extend higher in saddlebacks because of their higher anterior opening, we used already available data on neck length for all the tortoises (57 individuals) with domed and saddleback morphologies with available information from^15^, since neck length measures were not available for the 89 individuals from which we collected 3D data on the carapace.

### Estimate of the Center Of Mass (COM)

The center of mass (COM) of an object is a point that can be used as the location of the entire mass of the object, facilitating calculations in Newtonian physics. Since the distribution of internal organs makes the density of a tortoise non uniform, the COM could not be assumed to be in the geometric center of the shell. Therefore, to study self-righting potential in Galápagos giant tortoises using the 3D carapace reconstructions, we first need to determine the COM, which is currently not known for any turtle. We measured the COM in two live domed Galápagos giant tortoises at the Rotterdam Zoo – a male and a female. Assuming that the internal anatomy of saddleback and domed tortoises is not different, the position of the COM was assumed to be the same between the two shell morphotypes (but see Results).

**Figure 2:**
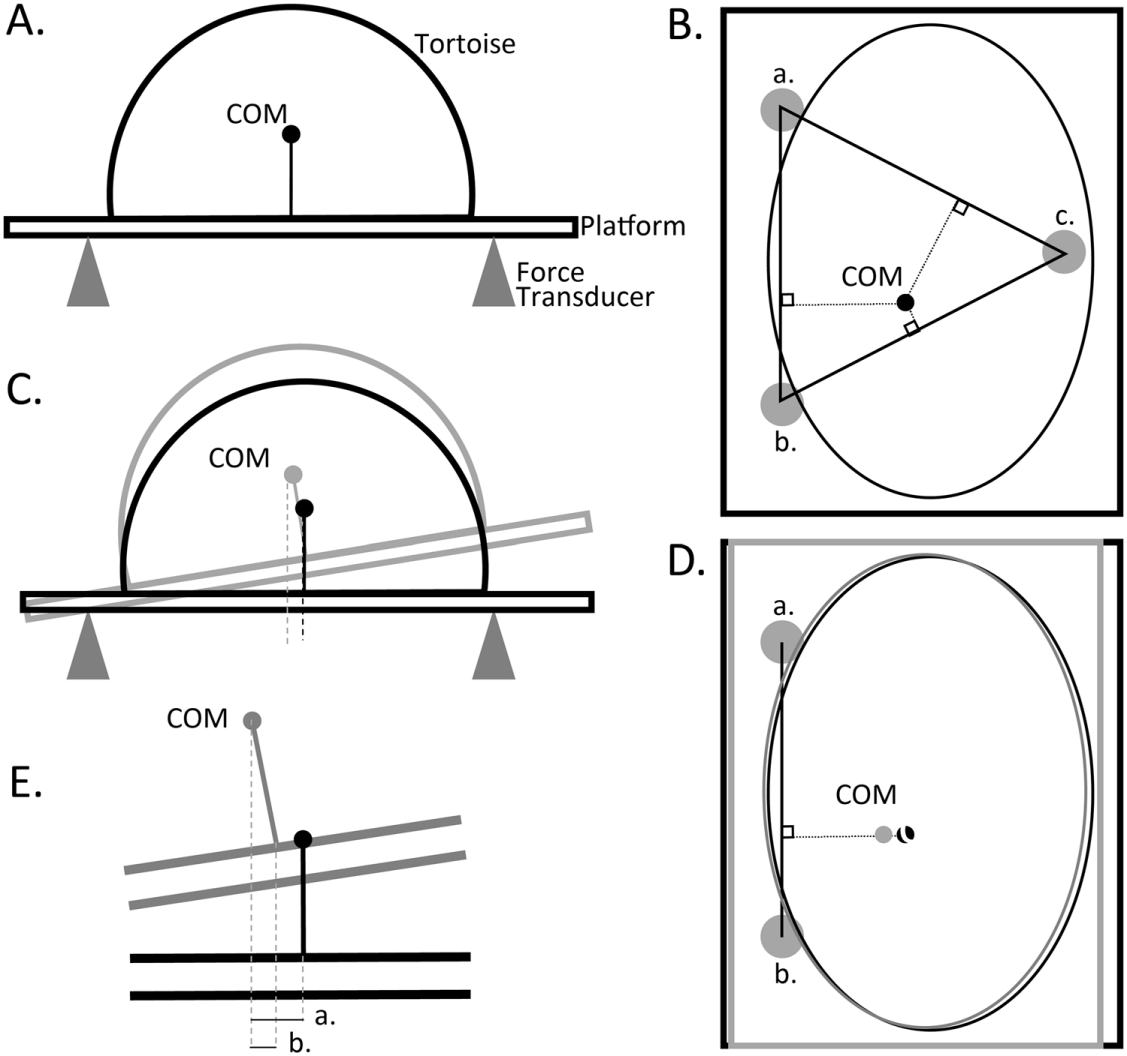
Schematic of the experimental approach used to calculate the COM. (**A**) Frontal view of the platform placed on three force transducers. (**B**) Dorsal view of the tortoise on the platform. Positions of the supporting force transducers (*a*., *b*., *c*.) and the COM are indicated. (**C**) The platform was tilted to allow measurement of the vertical position of the COM. Gray lines indicate the tilted platform. (**D**) Dorsal view showing the position of the tilted platform in gray. (**E**) Detail of the displacement of the COM: *a*. indicates the total horizontal displacement of the COM, *b*. shows the horizontal displacement due to the vertical position of the COM relative to the platform.

Each tortoise was placed centered on a platform supported by three force transducers (type Z6F C4 100 kg, HBM Benelux, Waalwijk, The Netherlands) at a distance of 75.3 cm from each other. All data from the force transducers were recorded at 20 Hz and subsequently filtered with a Bessel filter at 0.5 Hz before further use. During measurement, the tortoise did not move on the platform. The position of each tortoise relative to the platform and the force transducers was recorded by photographing the tortoise on the platform from several angles, and reconstructing landmarks on the tortoise and platform in 3D, using the PhotoModeler software. The total mass of the tortoise was recorded (see Results section), and the horizontal placement of the COM was calculated from the recorded force at each of the force transducers (Fig. 2A,B). Subsequently, the platform was tilted over a small angle on the long side. The tilted platform with the tortoise then rested on two force transducers and one unrecorded support point in the location of the third force transducer. The tilted platform caused the horizontal position of the COM relative to the force sensors to change (Fig. 2C,D). To remove the effect of the supporting plate, the COM of the plate was calculated to be in its geometric center, and the expected change of mass due to the plate at each angle was subtracted from the observed change in mass in force transducers *a* and *b*. The position of the COM was then recalculated from the corrected forces recorded at transducers *a* and *b* (Fig. 2). The apparent horizontal displacement of the COM was due to the displacement of the platform, as well as the vertical position of the COM (Fig. 2E). In order to correct the horizontal displacement of the platform, the horizontal displacement of the platform at the position of the COM (*b* in Fig. 2E) was calculated from the horizontal position of the COM and the tilt angle, and subtracted from the total horizontal displacement (*a* in Fig. 2E). The vertical aspect of the position of the COM (vertical in the frame of the non-tilted tortoise, Fig. 2) was then calculated from the tilt angle and the horizontal displacement. At least four tilted angles (max. 13.3°) were used to calculate the mean and standard deviation of the vertical position of the COM. The COM was thus calculated relative to the platform. The COM relative to the tortoise was then obtained by combining this data with the 3D reconstruction of the tortoise on the platform.

### Estimate of the Center Of Mass (COM) in the reconstructed tortoises

The position of the COM for each of the 89 reconstructed tortoises was estimated based on the 25 3D recorded landmarks (see above and Supplementary Table S1), using Generalized Procrustes Analysis (GPA). As in^12^, GPA was used to estimate the best transformation (including translation, orientation, and scale) minimizing the distance between two sets of landmarks from two tortoises. Using the optimal transformation, the reference COM was then warped. Different types of transformations were tested: (1) rigid, (2) rigid + global scaling (=similar transformation), and (3) rigid + scales + shear (=affine transformation). The rigid + global scaling was selected, as the rigid one only is unable to account for differences in turtle size, while the shear introduces unrealistic COM displacements. Knowing the COM for the two reference individuals, and since the results after GPA in terms of ratio (*h*_*max*_/*h*_*min*_−1, see below) were very similar if the male or female subject was used as the reference, the male subject was chosen to apply the estimated transformation to the COM to obtain the COM of the 89 measured tortoises.

The mass of each individual was estimated based on shell volume. The shell volume was computed using a triangle mesh reconstructed from the 25 landmarks. We assumed the total tortoise volume to be proportional to the shell volume, and that all tortoises have similar mass densities. The mass was therefore computed as *M* = V*Mr/*Vr*, where *V* is the shell volume, *Mr* is the mass of one of the reference tortoises, and *Vr* is its shell volume. For the two reference tortoise, densities Mr/Vr were close (3600 and 3450 kg.m^−3^).

### Data analysis

**Figure 3:**
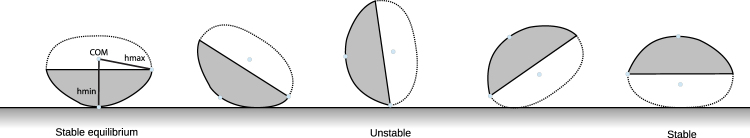
Schematic view of the self-righting movement of a tortoise shell, from a stable position with the animal overturned (upside down) to a stable position on its four feet. *h*_*min*_ and *h*_*max*_ are indicated in the figure, as well as the center of mass (COM).

To successfully self-right, a tortoise needs to provide an energy input that is higher than the energy deficit. The energy input may be given by pushing with the neck on the ground or by moving the limbs/head or by both^11^. The energy deficit – not including the energy due to friction or deformation of the soil or the shell - is likely due to the gravitational force that prevents self-righting (Fig. 3). We assumed the energy input to be proportional to both the mass of the animal (the heavier, the higher, see also Discussion) and the height of the shell. Thus, the energy input *E* is given by *E* = M * h_*min*_ * c, where *M* indicates the tortoise mass, *h*_*min*_ the distance between the center of mass and ground (before self-righting, Fig. 3), and *c* is an unknown parameter that depends on the momentum given by the neck or the waving of the head and legs or both (as in the models of^11^). The energy deficit is given by M * g * (h_*max*_ − *h*_*min*_), where *g* is the gravitational constant and *h*_*max*_ is the maximal COM-ground distance during self-righting (Fig. 3). Therefore, to successfully self-right the energy input must be higher than the energy deficit: M * h_*min*_ * c > M * g * (h_*max*_ − *h*_*min*_ *)*. Solving this equation results in a ratio of *c*/*g* > *(h*_*max*_/*h*_*min*_ − 1*)*, indicating that the higher the ratio *h*_*max*_/*h*_*min*_ − 1 is, the more difficult it will be for the animal to self-right, and thus higher input energy will be required. Due to how it is calculated, the ratio *h*_*max*_/*h*_*min*_ − 1 is independent from the mass of the animal. Following^11^, we expect that for saddleback tortoises, the energy input to roll-over mostly depends on the momentum given by the neck pushing on the ground and that the tortoise will successfully self-right if the neck length will be higher than *h*_*min*_. Therefore, we expect that saddleback tortoises will have higher *neck*/*h*_*min*_ than domed tortoises, with *neck* corresponding to the neck length, as this ratio determines if the head of the animal extends to reach the ground when the animal is upside down to give the push to overturn. Consequently, we compared *neck*/*carapace height* for domed and saddleback tortoises. Carapace height was used as a proxy for *h*_*min*_, which could not be calculated for the 57 museum tortoises for which data on neck length were available. Because neck length and carapace height may be differently influenced by the size of the animal, both measures (*neck* and *carapace height*) were first divided by carapace length, used as a proxy of size for each animal (Supplementary Table S1).

Before combining all the data from different species with the same shell morphotype for the analyses, we tested the influence of species assignment on self-righting potential taking into account sex differences and mass by using a two-way ANCOVA on the *h*_*max*_/*h*_*min*_ − 1, using the F-test and sum of squares with species, and sex as factors, and mass as a covariate (categories are unbalanced within each factor^28,29^). Since differences in self-righting potential among species with the same shell morphotypes were not observed (results not shown), data from different species were combined. Therefore, to study the significance of the relationship between self-righting, shell morphotype and sex, a two-way ANOVA was run on the *h*_*max*_/*h*_*min*_ − 1, using the F-test and sum of squares with shell morphotype (saddleback or domed) and sex as factors (categories are unbalanced within each factor^28,29^). Two – way ANOVAs were run to study the relationship between body mass and shell morphotype and between *neck*/*carapace height* and shell morphotype with shell morphotype and sex as factors. All the analyses were run in R v.3.3.1^30^.

## Results

### Estimate of COM (Center Of Mass)

The horizontal position of the COM was established from the force measurements with the platform in a horizontal position with small differences between values obtained from the three different combinations of transducers. Due to the small tilt angles used, the vertical aspect of the position of the COM was calculated with a larger standard deviation than the other two coordinates (Table 1). COM was located toward the ventral part of the animal in both turtles, independently of sex (Supplementary Fig. S2).

**Table 1:** Results of the measurements of the COM of the two live tortoises sampled at the Rotterdam Zoo. Position is given in a coordinate system relative to the platform. “F” and “M” indicate the female and male turtles, respectively.

Sex	Total mass (kg)	Coordinates COM (cm)		
		x	y	z
F	103.17	35.43 ± 5.02E-15	31.72 ± 2.5E-15	12.79 ± 1.92
M	133.90	43.44 ± 0.065	31.78 ± 0.065	8.91 ± 0.47

In our work, the position of the COM was assumed to be similar between saddleback and domed tortoises (see Materials and Methods). However, if the distribution of organ densities between the two shell morphotypes is different, assuming left/right symmetry of the animal, only the altitude of the COM would be influenced (vertical direction in Fig. 3). In the measure of self-righting potential (*m* = *h*_*max*_/*h*_*min*_ − 1), *h*_*max*_ will not change significantly, whereas *h*_*min*_ will be directly impacted by the vertical position of the COM. An error of *n%* on *hmin* will modify our measure as follows: *m*’ = *h*_*max*_/(*h*_*min*_ (1 + *n*/100)) −1, which is approximately (*m* − *n*/100), assuming that *n* is just a few percent. Our results indicate that the difference of (*h*_*max*_/*h*_*min*_ − 1) between domed and saddleback is about 0.05. Therefore, the difference in self-righting potential and the robustness of our results are significant if *h*_*min*_ is not under-evaluated by more than 5% for saddleback tortoises. It is unlikely that organ densities between closely related species can systematically increase *hmin* by more than 5%. Furthermore, since saddleback and domed shell morphotypes evolved multiple times across the archipelago, this would imply that differences in internal organ densities evolved multiple times in parallel with the shell for which all the domed tortoises have similar internal organ densities different from those of the saddleback tortoises. Therefore, we conclude that our measure of self-righting potential (*h*_*max*_/*h*_*min*_ − 1) is able to discriminate domed from saddleback morphotypes, if errors on COM estimate (due to different density distribution from the reference individual) does not exceed 5% of *h*_*min*_ (about 2 cm).

### Self-righting potential

Table 2 shows the results of the influence of sex and shell morphotype – domed and saddleback – on *h*_*max*_/*h*_*min*_ − 1 (used as indicator of energy deficit requirement) (Table 2a) and on neck length (Table 2c). Table 2b shows the results of the effect of shell morphotype difference on body mass. We found differences between the two shell morphotypes in energy deficit requirement (as a proxy for self-righting potential), body mass, and neck length (Table 2a–c), while we observed no difference in self-righting potential between females and males (Table 2a). Sexually mature domed tortoises have larger body masses than saddleback (difference in mass = 35.8 Kg, p-value < 0.001, Table 2b), with domed ranging from 17 to 327 Kg and saddleback from 15 to 112 Kg (Supplementary Table S1). Energy deficit is higher in domed tortoises than saddleback. The ratio *h*_*max*_/*h*_*min*_ − 1 is higher in saddleback tortoises by comparison to domed, implying that the first have more difficulty to self-right than the latter. Saddleback tortoises have a longer neck than domed (corrected neck length difference = 0.32, p-value «0.001), with males, independently of the shell morphotype, having slightly longer necks than females (corrected neck length difference = 0.15 inches).

**Table 2:** (a) Results of the two-way ANOVA on *h*_*max*_/*h*_*min*_ − 1, shell morphotype and sex in Galápagos tortoises. (b) Results of one-way ANOVA on the influence of shell morphotype on body mass in Galápagos tortoises. (c) Results of two-way ANOVA on the influence of shell morphotype and sex on neck length (see Materials and Methods) in Galápagos tortoises. Values in bold indicate significant p-values.

Category	Effect	*df*	Sum of squares	Mean square	F- value	p-value
(a)	Shell morphotype	1	0.04230	0.04230	41.616	≪**0**.**001**
	Sex	1	0.00026	0.00026	0.251	0.618
	Shell morphotype * sex	1	0.00395	0.00395	3.890	0.052
	*Residuals*	85	0.0864	0.00102		
(b)	Shell morphotype	1	26287	26287	13.240	**<0**.**001**
	*Residuals*	87	172671	1985		
(c)	Shell morphotype	1	1.2581	1.2581	67.469	≪**0**.**001**
	Sex	1	0.1936	0.1936	10.385	**0**.**002**
	Shell morphotype * sex	1	0.0062	0.0062	0.334	0.566
	*Residuals*	53	0.9883	0.0186		

## Discussion

We present the first data on individuals of Galápagos giant tortoises showing differences between saddleback and domed shell morphotypes in self-righting potential. Our approach allows assessment of the relative self-righting capacity of the two morphotypes, because it relies only on differences in shell morphotype, without confounding factors, such as the behavior of individual animals, which could be more or less active, or the contribution of the legs and neck to self-righting. Overall, our results support the hypothesis of^11^ that tortoises with a less rounded shell shape (saddleback morphotype) may use their neck to create a momentum to self-right. Our data indicate in fact that saddleback tortoises have higher ratio of neck length versus shell height. Furthermore, our work develops a simplified and qualitative model of the energy input needed to successfully self-right. Specifically, in comparison to previous studies focusing on the relationship between self-righting and shell morphology (*e*.*g*.,^11,12^), we used 3D reconstructions of real individual shells instead of simplified curves, therefore improving the accuracy of the height of the shell and its curvature. We also experimentally estimated the COM for two living individuals of Galápagos giant tortoises. This approach allows to more correctly estimating where the forces acting on the body in motion are applied, and consequently the parameters (*h*_*max*_ and *h*_*min*_ instead of carapace height and width) of relevance to properly assess self-righting ability in the studied individuals.

Our results show that, based only on shell morphotype, saddleback tortoises require a higher energy input than domed ones to successfully self-right. In most tortoise species, overturning generally occurs as a result of male-male agonistic behavior to establish dominance (*e*.*g*.,^4^) or falling due to locomotion on uneven grounds or falling down from sloped surfaces^8^. Agonistic behavior is unlikely to be the most common factor causing overturning in Galápagos giant tortoises, as individual competition occurs in this species by vertical extension of the head^25^ and male – male competition in wild animals does not occur often (E. Garcia, pers. comm.). However, the uneven terrain consisting of lava rocks, especially in the drier parts of the islands, makes stable locomotion particularly difficult and tortoise overturning occurs. Galápagos giant tortoises are known to falling among the lava rocks and inefficient self-righting is considered the most common cause of natural death for the adult^31^ (E. Garcia, pers. comm.). Selective pressure toward improved self-righting performance could therefore drive morphological evolution.

Our results indicate that saddleback tortoises may self-right by vertically pushing the head on the ground and then by bobbing their feet, while domed tortoises rely on moving their feet and head to gain sufficient momentum to self-right^11^. The higher anterior opening of the saddleback shell and the smaller size of these animals would provide the higher energy input required to self-right through the longer neck (this work) and longer extension of the neck^25^. The overall smaller body mass of saddleback tortoises compared to domed ones would also allow lowering the required energy input (due to overall lower energy deficit). Since mass and muscular force do not scale isometrically, for smaller individuals using muscular force applied directly to the ground to self-right may be at an advantage. Assuming isometry, larger individuals which use momentum from flailing extremities may experience less advantage of a smaller size, as this would also reduce the mass of the extremities, limiting the momentum that can be gained by flailing.

All the proposed hypotheses to explain the adaptive value of the different shell morphotypes observed in Galápagos giant tortoises - different use of feeding resources, thermal adaptation (temperature-size rule^32^), and self-righting – stem from the observed correlation of each shell morphotype inhabiting a specific habitat type (drier for saddleback tortoises, mesic for the domed ones). These hypotheses are not mutually exclusive, and both adaptation and exaptation (a trait of the organism that was not select for that role, but that improves fitness^33^) most likely occur. If, for example, the different shell morphotypes evolved primarily to optimize self-righting once the animals fell on their back (adaptation), the smaller body size, the higher anterior opening and longer neck of saddleback tortoises could also have improved their fitness in terms of thermoregulation and using different feeding resources (exaptation).

To our knowledge, none of the hypotheses relative to selection and adaptation of distinct Galápagos giant tortoises shell morphotypes have been tested previously on wild individuals from multiple populations. The scientific literature mostly supports the hypothesis that the two shell morphotypes are adaptive for feeding on different resources in the two environments (*e*.*g*.,^18–23^). In drier environments, rich vegetation is scarcer than at higher elevations and the pads of the different species of giant prickly – pear *Opuntia* (*Opuntia* spp.) cacti can become an important food source for the saddleback tortoises^23^. The higher anterior opening of the shell of saddleback tortoises facilitates raising the neck higher than domed tortoises can, and could therefore have evolved to allow the animals to use this source of food^17–19^. However, this seems unlikely, as saddleback tortoises currently only seldom feed on *Opuntia* tree^25,34^. However, this may have been a much more important food resource for saddleback during their evolution. Field observations indicate that saddlebacks feed primarily on *Opuntia* when other vegetation is scarce (*e*.*g*., years of drought, for example during La Niña or especially dry seasons^35^), while most of time, as in many other tortoises, they eat any available plants and fruits^25,34,36^ (but see^23^).

Our work represents the first instance in which it has been shown that the two different shell morphotypes differ in the energy required to potentially self-right, a fitness – related function, which may therefore be related to shell shape evolution in Galápagos giant tortoises. Our results indicate a lower self-righting potential in saddleback as compared to domed tortoises based on shell shape. The longer necks and the higher neck extension due to the anterior opening of saddleback tortoises could possibly provide higher energy input for self-turning than in domed tortoises. These results do not demonstrate *per se* that self-righting efficiency was the selective pressure acting on shell shape variation in Galápagos giant tortoises. From the data alone, we cannot establish what evolved first in the saddleback morphotype: the longer and higher extension of the neck or the shell shape. A longer and higher extension of the neck could have relaxed the selective pressure on the shell shape, which became smaller and less rounded than in domed tortoises, as self-righting efficiency was mostly achieved by the vertical pushing of the neck on the ground.

On the other hand, if for whatever reason the overall saddleback shell shape, which is also smaller in size and more compressed laterally than the domed one, evolved before the evolution of longer neck and the higher anterior opening, selection for improved self-righting performance could have also pushed for the evolution of increased neck lengths and a higher anterior opening in saddleback animals. Although the presence of similar morphologies occurring in similar environments and evolving multiple times across a phylogenetic tree – as in the case of the Galápagos giant tortoise shell morphotypes – has been long interpreted as adaptive, this phenomenon could occur for other reasons than adaptation^37^. Only direct experimental measure of functional performance and selection strength could validate the adaptive value of phenotypic trait and thus allow distinguishing between adaptation and exaptation^33,37^. However, measuring selection in this system in particular is impossible and in general very difficult to do in most cases, as it would require recreating the conditions in which the trait evolved. On the other hand, studying the functional performance of traits and indirectly relating it to its potential influence on fitness, as in this work, could provide important insight in the adaptive value of traits. Although empirical fitness data would be required to properly assess the adaptive value of each shell morphotype for self-righting, these data are very difficult to collect in long living organisms as the Galápagos giant tortoises. Further functional comparative analyses on thermal ecology, behavioral ecology, feeding ecology, and agonistic behavior on saddleback and domed tortoises, will provide additional data to understand adaptation and exaptation in shell shape in Galápagos giant tortoises.

### Data availability

A video-abstract for this paper can be viewed using the following link https://youtu.be/8MNNjNKWVuc. Landmarks of the carapace 3D reconstructions for all the 89 individuals used in this study, individual body mass, data on *h*_*max*_/*h*_*min*_ − 1, energy deficit, and neck data are provided as Supplementary Table S1. Landmarks of the carapace 3D reconstructions for the tortoises sampled at the California Academy of Sciences (CAS) will be deposited at the CAS and associated to the tortoise museum voucher.

## Electronic supplementary material

Supplementary Data

COM position
