# Supplementary material for: Self-righting potential and the evolution of shell shape in Galápagos tortoises
Source: Sci Rep. 2017 Nov 30;7:15828. doi: 10.1038/s41598-017-15787-7 (PMC5709378; doi:10.1038/s41598-017-15787-7)

Self-righting potential and the evolution of shell shape in Galápagos tortoises

Ylenia Chiari, Arie van der Meijden, Adalgisa Caccone, Julien Claude, Benjamin Gilles

Supplementary Information 2. 3D reconstruction of one of the turtles sampled at Rotterdam zoo with the relative position of the COM.

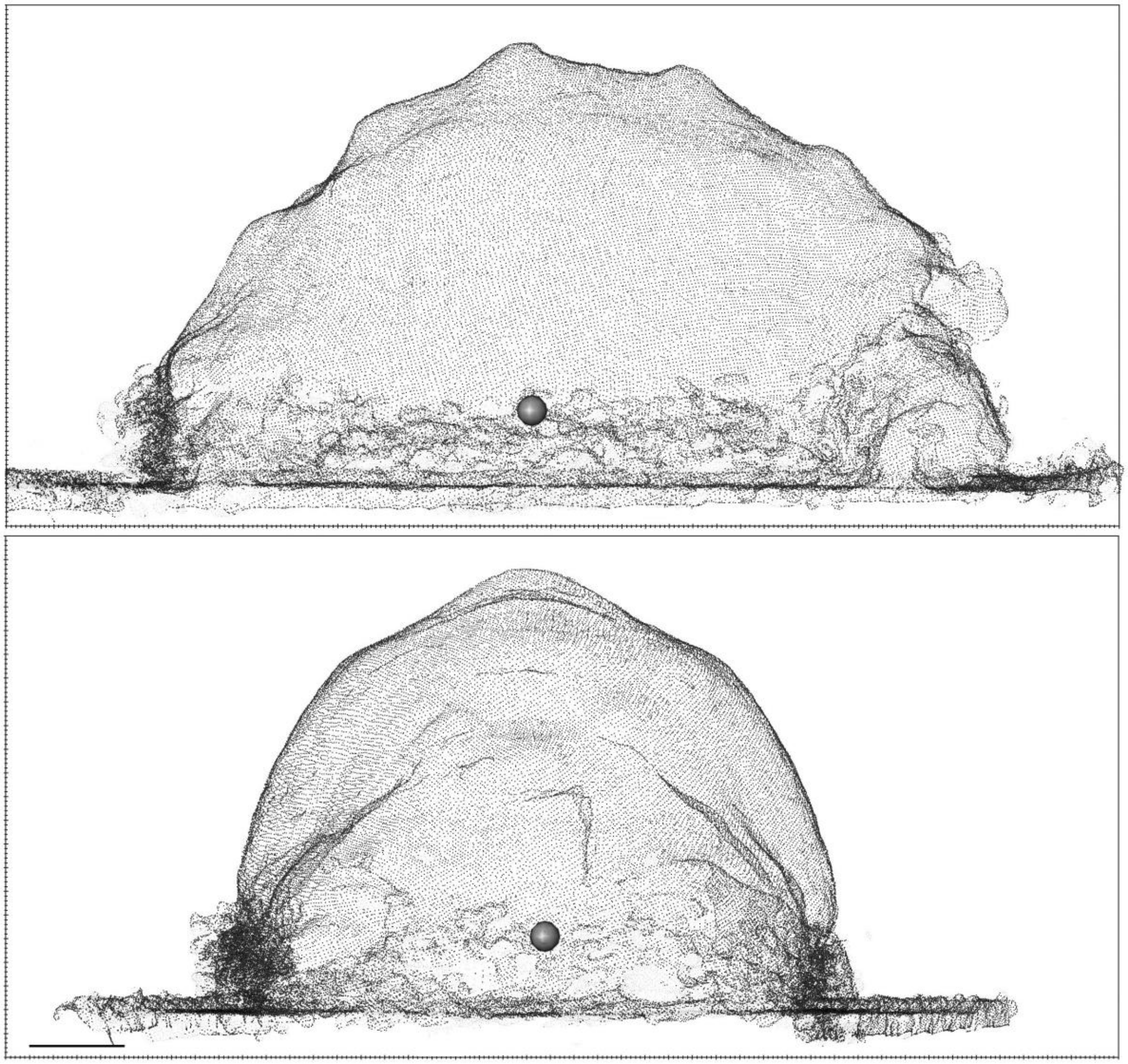

Supplement: Supplementary file 2 — COM position [file 41598_2017_15787_MOESM2_ESM.pdf]
